# *Foods*: Where Innovation, Agriculture, Molecular Biosciences and Human Nutrition Meet 

**DOI:** 10.3390/foods1010001

**Published:** 2012-11-21

**Authors:** Charles Brennan

**Affiliations:** Department of Wine, Food and Molecular Biosciences, Lincoln University, Lincoln 7647, New Zealand; Email: Charles.brennan@lincoln.ac.nz; Tel.: +64-3-321-8236.

There is one commodity the world over that unites mankind—food. In 2011 the United Nations claimed that the world’s population had reached the seven billion mark, a number which is set to increase dramatically in the decades to come. Food security, supply and sustainability are of paramount concern to the future economic and social progress of humanity. It is the responsibility of the food industry, together with food scientists and technologists, to shoulder the burden of ensuring an adequate supply of nutritious, safe and sensorially acceptable foods for a range of demanding consumers. In responding to this challenge, we need to understand the link between agriculture, engineering, food processing, molecular biosciences, human nutrition, commercialisation and innovation. Access to information concerning the composition and quality of foods has never been so easy for consumers and technologists alike. A plethora of research publications are made available each month to scientists and associated interested parties. The outcomes of these research manuscripts are often distilled and disseminated into messages available to everyone through bulletin boards, forums and the popular press. Newspapers and new agencies constantly report on the latest pharma-medical finding, or news regarding food safety and security concerns. We live in an age where information is so readily available to everyone that the task of finding credible and reputable data can be difficult at times. Providing sound evidenced based research is where a peer-reviewed journal can provide clarity.

It gives me great pleasure therefore to welcome you to our new open access journal called *Foods*. The title of the journal is deliberately broad to reflect the complexity of scientific approaches employed by researchers in order to create critically evaluated articles which advance our understanding of not only food science and technology, but all the areas of importance around the whole food system. This is where we aim to differ from journals which have developed their reputation on specific elements of food (for instance food chemistry, biochemistry, consumer preference, food quality, food technology). *Foods* aims to encompass all of these areas and more so that the connections between food production, processing, microbiological stability and consumption are related to food structure, human nutrition, consumer preference and food legislation. This mix of sciences and technologies can be observed by the varied elements covered in the inaugural issue of our Journal. 

There is no question that agricultural practices are of significant importance when considering both the sustainability of food production and also food security in ensuring the quality and quantity of adequate raw ingredients. Malnutrition is a challenge the world faces not solely because of lack of produce, but also from the inefficiencies in supply chain dynamics and manufacturing technologies. Science, commerce and innovation are all required to resolve these issues. If we can develop ways to simplify supply networks and ensure process and production optimisation by minimizing waste and energy consumption, many social and environmental issues could be resolved. 

Researchers are constantly striving to improve crop yield or quality through a range of conventional and modern breeding techniques. Optimistically we can point to harnessing the potential benefits of proteomics and genomic technology as helping us to constantly improve pre-farm gate production. During the last 20–30 years the food industry has also provided extraordinary innovation in engineering practices post-farm gate. These improvements allow for consistent quality of microbiologically stable food items. In turn, the innovations in the understanding of chemical and biochemical principles of food composition and processing have created new knowledge streams in terms of functional bioactive ingredients with which we have the potential to manipulate the nutritional profile of populations. My own small area of research—the understanding of phytochemicals and how by manipulating food processing conditions we can manipulate the nutritional quality of foods such as glycaemic response, mineral availability or phytochemical bioavailability—is a relevant example of how science and innovation can lead to considerable beneficial developments for both the consumer and the industry. In the years to come I hope that this journal will aid in the promotion of scientific understanding by publishing high quality outputs from dedicated scientists and, being an open access journal, ensuring that this information will be freely available to anyone.

So my considerable thanks go to MDPI publishers and our publishing team for supporting this new initiative. My thanks also go out to my illustrious editorial board without whom this journal would struggle to exist. The complexity and breadth of interest and expertise amongst the editorial board is outstanding and with their help we will endeavour to ensure that *Foods* contributes to the improvement of knowledge transfer throughout the world. 

*Foods* will publish high quality reviews, research papers, communications and short notes. Submissions are welcome in subject areas relevant to the aims and scope of the journal. Our aim is to facilitate the publication of applied and theoretical research in as much depth as is required. Manuscripts will be peer-reviewed by members of the editorial board and invited reviewers and we seek to offer a quick submission-review-publication process of between 4 and 6 weeks.

I look forward to receiving and reading your submissions and trust that with your help we can make *Foods* a preeminent journal in the areas of food science, technology and nutrition.

